# Evaluation of using the Anderson-Montesano and the Tuli classifications in pediatric patients with occipital condyle fractures

**DOI:** 10.1186/s13018-021-02463-w

**Published:** 2021-07-13

**Authors:** Ryszard Tomaszewski, Jacek Kler, Karol Pethe, Agnieszka Zachurzok

**Affiliations:** 1 Department of Pediatric Traumatology and Orthopedy, Upper Silesian Child Centre in Katowice, 40-752 Katowice ul. Medyków, 16 Katowice, Poland; 2 Faculty of Science and Technology, Institute of Biomedical Engineering, University of Silesia, Katowice, Poland; 3 Department of Pediatrics and Pediatric Endocrinology, School of Medicine in Katowice, Medical University of Silesia, Katowice, Poland

**Keywords:** Occipital condyle fracture (OCF), Children, Treatment, Classifications, Cranio-cervical junction (CCJ)

## Abstract

**Background:**

Occipital condyle fractures (OCFs) in patients before 18 years of age are rare. Classifications of OCF are based on the CT images of the cranio-cervical junction (CCJ) and MRI. The Anderson-Montesano and Tuli classifications are the types which are most commonly used in these cases. Classification of OCFs allows the implementation of OCF treatment. The aim of this study was to evaluate the effectiveness of using the OCF classification in pediatric patients based on the analysis of our own cases.

**Methods:**

During the years 2013–2020, 6 pediatric patients with OCFs, aged 14–18, have been treated. Two patients with unstable fracture III according to Anderson-Montesano and IIB according to Tuli were treated with the halo-vest. Additionally, one patient presenting neurological symptoms and with an associated C1 fracture was qualified for the halo-vest stabilization as well. The other patients were treated with a Minerva collar. We evaluated the results 6 months after completing the OCF treatment using the Neck Disability Index (NDI) and SF-36 questionnaires. Confidence intervals for the mean values were verified using the MeanCI function (from the R library DescTools) for both classical and bootstrap methods.

**Results:**

Based on NDI results, we have obtained in our patients an average of 4.33/45 points (2–11) and 9.62% (4.4–24.4). Based on the SF-36 questionnaire, we obtained an average of 88.62% (47.41–99.44).

**Conclusion:**

The Anderson-Montesano and Tuli’s classifications of OCF can be used to assess the stability of OCF in adolescents, but both classifications should be used simultaneously. CT and MR imaging should be used in diagnosing OCFs, whereas CT allows assessing therapeutic outcomes in OCF.

## Background

Occipital condyle fractures (OCFs) in patients before 18 years of age are rare, accounting for about 1–3% of all cervical spine injuries [1, 2]. This fracture usually accompanies high-energy injuries, especially traffic trauma with associated head injuries. Due to the often coexisting polytrauma and what is related to this a complex radiological diagnosis, the number of patients diagnosed with OCF in recent years has increased, especially before the age of 18 years [3]. Classifications of OCF are based on the CT images of the cranio-cervical junction (CCJ) and MRI [2]. The Anderson-Montesano and Tuli classifications are the types which are most commonly used in these cases [1, 4–7].

Classification of OCFs allows the implementation of conservative OCF treatment based on the immobilization of the patient with a hard collar or cervical orthosis as well as surgical treatment by using the halo-vest, open reduction internal fixation (ORIF), or an occipito-cervical fusion [3, 8, 9].

The aim of this study was to evaluate the effectiveness of using the OCF classification in pediatric patients based on the analysis of our own cases. The authors’ hypothesis was that the Anderson-Montesano and Tuli classifications could be effectively used in pediatric patients by taking into account the specificity of pediatric fractures. Therefore, the authors have observed elements which are non-specific for children in both of these classifications.

## Materials and methods

During the years 2013–2020, 6 pediatric patients with OCF, aged 14–18, have been treated in our department. The mean age of the patients was 15.8. There were 3 females and 3 males among these pediatric patients. After admitting the child to the emergency department, each patient was examined by a pediatric team consisting of a pediatrician, a pediatric surgeon, a neurologist, and an orthopedic surgeon.

**Fig. 1:**
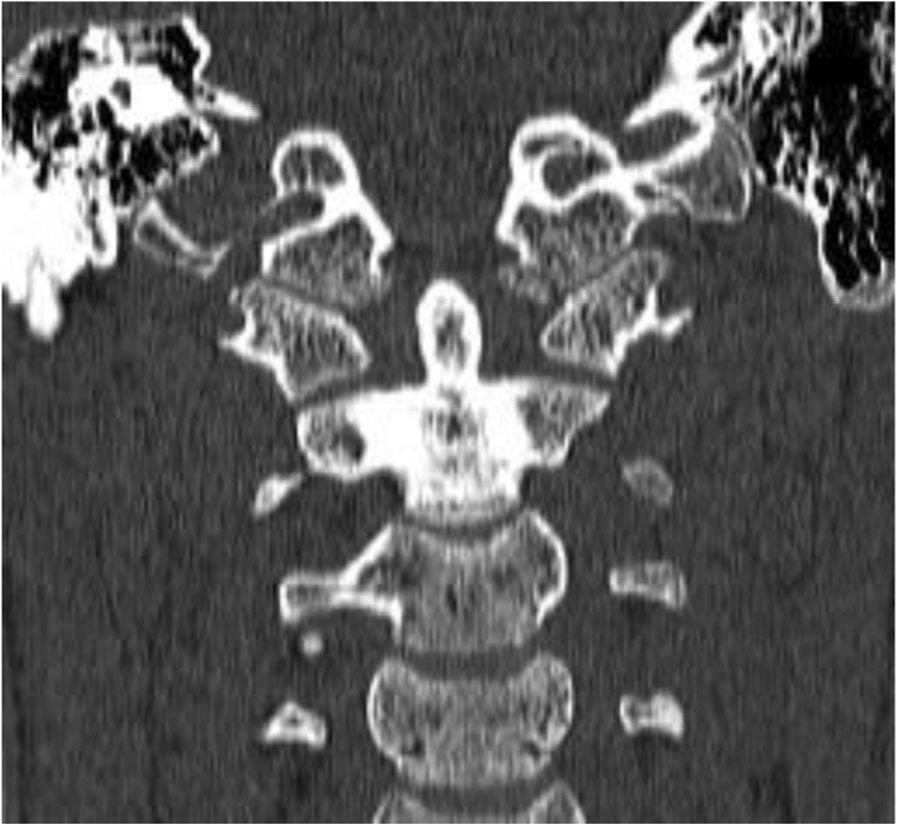
Patient, 16.1 years old. The cause of the trauma was fall from a bike. OCF classified as type I according to Anderson-Montesano and IIA according to Tuli

**Fig. 2:**
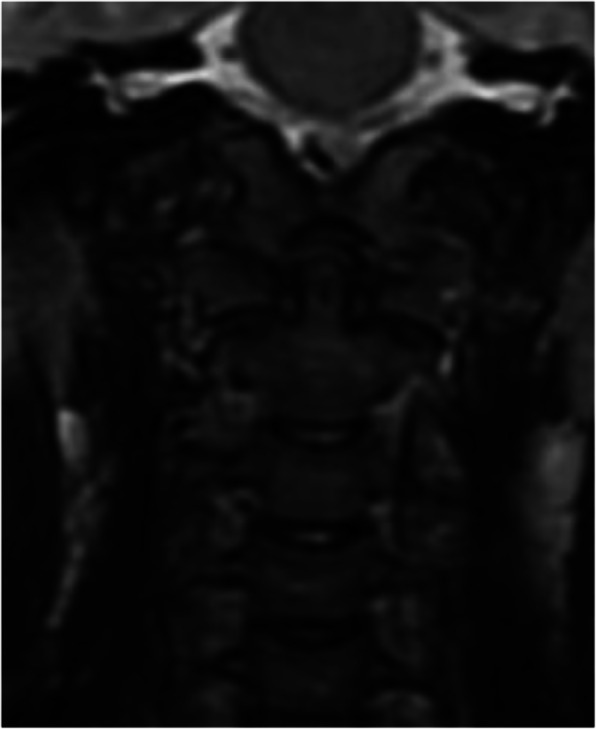
Patient from Fig. 1. MR after injury

**Fig. 3:**
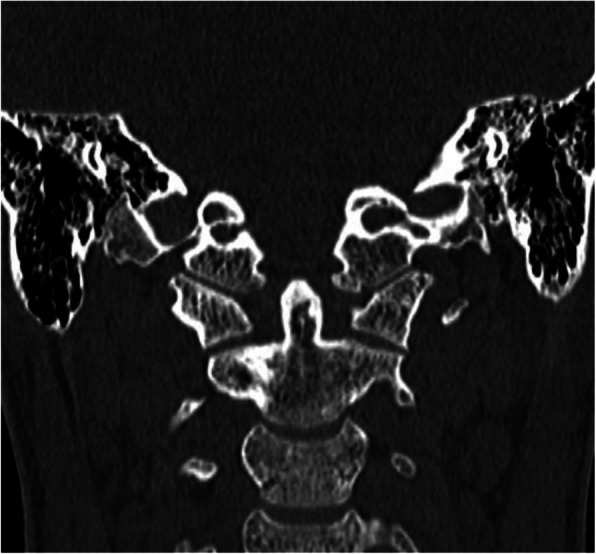
Patient from Figs. 1 and 2. CT performed 5 months after the accident. Treatment—rigid neck collar for 12 weeks

The majority of patients (3 patients) were injured in motor vehicle accidents (these patients were car passengers). One patient was stroke by a car, one patient fell from a bicycle, and one patient was injured by falling from a height. All patients had head injuries, including 4 cranial bone fractures considering the frontal or facial bones and 5 intracranial injuries. Two patients sustained thoracic injuries with pulmonary contusion or pneumothorax. Accompanying cervical spine fractures were observed in 2 patients; also, two patients presented upper extremity fractures. There were no patients with bilateral OCF (Table 1) All patients underwent a CT trauma scan and subsequent MR imaging to visualize the ligament damage. All the patients underwent neurological examination for possible nervous system lesions and additional consultations according to the associated injuries. After the CT scan analysis using the Anderson-Montesano and Tuli classifications, the patients were qualified for further therapeutic management. Two patients with unstable fracture III according to Anderson-Montesano and IIB according to Tuli were treated with the halo-vest (Figs. 1 and 2). Additionally, one patient presenting neurological symptoms and with an associated C1 fracture was qualified for the halo-vest stabilization as well. The other patients were treated with a Minerva collar. The halo-vest stabilization was maintained for 13.1 weeks (12.5–14) and a cervical collar for 11.3 weeks (11–12). During the treatment, the patients were monitored by CT or MR imaging. The group of patients treated with the halo-vest was monitored for proper correction by CT examination 1 day after the halo-vest placement (Fig. 3). After confirming the satisfactory alignment of the injured bone fragments, the patients were discharged from the unit with the recommendation of a conscientious lifestyle and observation for the occurrence of distressing symptoms.

**Table 1:** Patients records and time of treatment

Name	Sex	Age	Anderson-Montesano	Tuli	Cause of injury	Trauma accompanying	Halo-vest time of treatment	Minerva brace
P.P	M	15.2	III-unstable	IIB	Traffic accident—car passenger	Left frontal fracture, left frontal sinus fracture, fracture of the left frontal bone, contusion of the frontal lobe of the brain	12.5 weeks	–
K.D.	F	15	III-unstable	IIB	Pedestrian hit by a car	Contusion of the right lung, concussion of the brain, multiple abrasions of the epidermis	13 weeks superficial pin infection	–
R.M	F	18	I	IIB	Traffic accident—car passenger	Post-traumatic aphasia, pneumothorax, pyramidal-posterior paresis	14 weeks	–
S.D	M	14.7	III-stable	IIA	Traffic accident—car passenger	Right frontal bone fracture, nasal bone fracture, subdural hematoma	–	12 weeks
B.W	F	16	I-stable	IIA	Fall from a height	Right frontal bone fracture, nasal bone fracture, subarachnoid bleeding, Th3-5 transverse process fracture, fracture of the proximal epiphysis of the radial bone	–	11 weeks
M.O	M	16.1	I-stable	IIA	Bike ride fall	Left frontal bone fracture, right maxillary sinus fracture, right orbit fracture, V metacarpal bone fracture	–	12 weeks

The CT of cranio-cervical junction (CCJ) was performed in all patients at the end of the treatment, in patients treated with the halo-vest after 13 weeks (11–14), and with the cervical collar after 11 (9–12) weeks.

The authors with a group of radiologists (2) and orthopedic surgeons (2) evaluated the CT and MRI findings of the CCJ performed immediately after injury and after achieving the OCF bone consolidation. In the group of patients with unstable OCFs based on the Anderson-Montesano classification in two patients (patient initials: P.P., K.D), we have classified fractures as type III and according to Tuli’s classification as type IIB, while in one patient (R.M.), OCF was assessed as type I according to the Anderson-Montesano classification. Based on Tuli’s classification, in which the C0–2 complex is assessed due to the occurrence of C1 fracture, we found the presence of an unstable OCF. In the remaining three patients evaluating CT and MRI’s of the CCJ, the authors have observed (in patients S.D., B.W., M.O.) OCF type I according to the Anderson-Montesano classification and IIA according to the Tuli classification. During the evaluation of OCFs, there was a full consensus among the four team members. Still, it should be noted that there were difficulties in evaluating and differentiating between type I and III, according to the Anderson-Montesano classification.

The authors have also evaluated the C0–2 ligamentous apparatus based on CCJ MRI. We have noted the lack of damage to the C0–2 ligamentous apparatus in our group with particular attention to the ligament alar, apical, and tectoral membrane.

According to the Anderson-Montesano classification, in patients with type III OCF, we have observed a 10 and 12° rotational displacement in the C0–1 joint, as well as a 3- and 4-mm displacement in this joint. According to the Anderson-Montesano classification, in other patients with type I OCF, we found no rotational or translational displacement in the C0–1 joint.

We evaluated the results 6 months after completing the OCF treatment using the NDI and SF-36 questionnaires. The NDI questionnaire was modified due to the age of our patients by removing item 8 regarding driving. In each case, the questionnaire was completed by the patient himself but assisted by one of the parents. In one patient (patient initials: R.M.), we asked one of the parents to complete the questionnaire in case of difficulty answering the questions independently.

## Results

The follow-up was 4.5 years (1–7). The patients’ age in our series of patients is noteworthy—mean age 15.8. These are children after puberty.

We also evaluated the results of the treatment based on the CT of the CCJ performed at the end of the therapy. In all of our patients, bone consolidation was obtained, but in patient P.P., the presence of displacement of the occipital condyle bone fragment by 1 mm and rotation of 0° degrees was observed. In other patients, bone fusion was obtained without necessary CT evaluation of displacement of bone fragments of the occipital condyle and lack of rotation in the joint C0–1. This, in our opinion, confirms the classifying of OCF Anderson-Montesano, with OCF instability in type III, which did not occur in our patients after they had achieved bone consolidation.

Based on the Neck Disability Index (NDI) results, we have obtained in our patients an average of 4.33/45 points (2–11) and 9.62% (4.4–24.4).(Tables 2 and 3). It should be noted that our patients had intermittent CCJ pain (1.166 points) and headaches (0.833 points) after ending the treatment. Based on the SF-36 questionnaire, we obtained an average of 88.62% (47.41–99.44) (Table 4). However, patients based on the questionnaire assessed health change in 83.33% and social functioning in 85.4%, while emotional well-being in 93.33%, energy fatigue in 92.5%, and physical functioning in 90.83%.

**Table 2:** Mean values calculated as the sum of scores (points for NDI and percent scale for SF-36) divided by the number of questions (9 for both surveys) of responses of individual patients in NDI and SF-36 questionnaires

Patient	NDI		SF-36
	Mean individual score	Individual summaric score per 45	Mean [%]
P.P	0.3333333	3	96.94444
K.D	0.3333333	3	90.46667
R.M	1.2222222	11	47.41111
S.D	0.2222222	2	99.44444
B.W	0.5555556	5	98.05556
M.O	0.2222222	2	99.44444

**Table 3:** Mean values of quantifiers used to respond in the NDI questionnaire. The last column of the table contains the number of answers with a non-zero value for the particular quantifier

Quantifier of the NDI questionnaire	Mean	No. of instances in the survey
Pain intensity	1.1666667	6
Personal care	0.1666667	1
Lifting	0.1666667	1
Reading	0.5000000	3
Headaches	0.8333333	4
Concentration	0.3333333	2
Work	0.1666667	1
Sleeping	0.8333333	5
Recreation	0.1666667	1

**Table 4:** Mean values of quantifiers used to respond in the SF-36 questionnaire

Quantifier of the SF-36 questionnaire	Mean [%]
Physical functioning	90.83333
Role limitations due to physical health	87.50000
Role limitations due to emotional problems	88.90000
Energy fatigue	92.50000
Emotional well-being	93.33333
Social functioning	85.41667
Pain	89.16667
General health	86.66667
Health change	83.33333

## Discussion

The most important finding of this study is that the Anderson-Montesano and Tuli classifications of OCF can be used in adolescents during and after puberty, and these classifications are useful for the treatment for OCF based on the CCJ. However, we do not make recommendations for the use of these classifications in pre-adolescent patients due to the lack of such patients in our patient series and the difficult interpretation based on the bibliography of pre-adolescent OCF, as it is based primarily on case reports and often lacks a detailed description of radiologic studies, including CT and MRI, making objective radiologic evaluation and subsequent qualification for the treatment of these patients impossible [5, 10].

OCFs are usually the result of high-energy trauma [4, 11], especially road traffic injuries, as confirmed by our patient series. It is worth noting that the second cause of OCFs in our patients was extreme sports accidents. OCF can be fatal or often diagnosed post mortem. Bell made the first description of OCF in 1817 during a post mortem examination [1, 12–14].

In all of our patients, head trauma and cervical spine injuries were found during the examination in the emergency department. Therefore, one of the examinations which was performed after admitting our patients was CT imaging of the head and cervical spine. CT is the examination of choice in the diagnosis of OCF because of its excellent imaging of bony structures of the CCJ [15]. CT’s role as an essential examination in the diagnosis of OCF was emphasized by Kruger et al. [4]. Maserati [9] confirms the necessity of urgent CT of the cervical spine for OCF diagnosis; however, Bloom [16] also emphasizes to focus on the patient’s clinical condition as well, which, according to the author, has a significant impact on the time of CT of the CCJ. We also performed CCJ MRI due to OCF in all of our patients within a 24-h period from admission. Aulino [6], in a series of 76 patients with OCF, performed MRI in all patients within 2 weeks, basing the primary diagnosis on CT of the head and cervical spine performed after injury. However, Bystrom [17] and Roy [2] point out the possibility of evaluating the ligamentous apparatus in OCF only basing on the MRI. Tat [18] evaluated the spinal cord and the soft tissues of the CCJ in pediatric patients with MRI. He also emphasized the longer examination time of MRI compared to CT and the possible need for general anesthesia of the patient.

In our patient’s series, we used CT to evaluate OCF repositioning after the halo-vest placement and after achieving OCF bone fusion. This approach is also confirmed by Hanson [3] and Leone [19] using CT in the final evaluation of OCF treatment.

The authors did not perform radiographic imaging (XR) because of OCF, because the evaluation of OCF on the basis of XR is unavailable due to the difficulty in achieving good PA and lateral projection of the occipital condyles, primarily because of the overlapping mandible which covers these elements. The projection through the “open mouth” is not very useful as well because it only partially shows the occipital condyles and is difficult to perform in pediatric patients especially immediately after trauma [3]. Some authors point out the presence of accompanying OCF swelling of the paravertebral soft tissues, but this symptom is difficult to interpret in objective radiological evaluation [6, 15, 20]. However, he points out that many authors when presenting OCF in pediatric patients performed radiographic imaging as the first test in their patients. Aulino [6] points out conventional cervical spine radiographs were available for review in 60 of the 76 patients. The OCF was not visible by radiography on any of these patients; although skull fracture of the cranial base was often observed; however, direct involvement of the occipital condyle was not visible. Furthermore, the lack of XR-based OCF classifications is noteworthy.

Currently, the Anderson-Montesano and Tuli classifications are commonly used in OCF assessment [1]. Anderson and Montesano in 1988 [21] divided the OCF into 3 types according to the mechanism of injury: type 1 arises as a result of an axial force and presents as a fracture of the condyle without displacement, and it is a stable fracture; type 2 arises as an extension of the occipital bone fracture gap, which passes to the skull posture, results from direct trauma to the skull, and is a stable fracture as well; and type 3 is an avulsion fracture; the condyle fragment becomes detached by the alar ligament due to rotational injury and is an unstable fracture. Tuli, in 1997 [22], presented a new classification noting that types 1 and 2 according to A/M are treated using the same method; therefore, this distinction does not bring new information. Tuli divided OCF into type 1—stable fracture without displacement—and type 2, which has subtypes A and B. Type 1 requires no specific treatment, type 2A can be treated with a rigid cervical collar, and type 2B requires halo or surgical treatment. The criteria for classifying OCF into types 2A/2B are based on the demonstration of signs of instability of the 0-C1-C2 complex on imaging studies. Type 2A is a displaced fracture without signs of 0-C1-C2 instability; type 2B contains at least one instability criteria. CT scan and/or X-ray criteria of 0-C1-C2 instability: >8° of axial rotation of O-Cl to one side, >1 mm of O-Cl translation, > 7 mm of the overhang of Cl on C2, >45° of axial rotation of C1-C2 to one side, > 4 mm of C1-C2 translation, <13 mm distance between the posterior body of C2 to the posterior ring of Cl, and avulsed transverse ligament with MR evidence of ligamentous disruption.

Mueller [8] in 2011 proposed his own classification of OCF in which he distinguished 3 types: type 1 is unilateral OCF without atlanto-occipital dislocation (AOD), type 2 meaning bilateral OCF without AOD, and type 3 which is unilateral or bilateral OCF with AOD. This classification has not been more widely used in the treatment planned for OCFs.

It should be noted that the CCJ is often functionally evaluated as one mobile complex, which is included in the Tuli classification. The CCJ consists of the paired atlanto-occipital joints, the anterior and posterior median atlanto-odontoid joints, and the paired atlanto-axial joints [19]. The cranio-cervical ligamentous anatomy can be divided into two groups meaning the intrinsic and extrinsic ligaments [12]. The extrinsic ligaments consist of the anterior and posterior atlanto-occipital ligaments, the articular capsule ligaments, and 2 lateral atlanto-occipital ligaments. The intrinsic ligaments, which lie within the spinal canal provide most of the ligamentous stability. From anterior to posterior, they are the odontoid ligaments (alar and apical), the longitudinal band of the cruciate ligament, and the tectorial membrane. Cranio-cervical flexion is limited by the bone anatomy, while the tectorial membrane limits the extension. The contralateral alar ligaments restrict rotation and lateral flexion, and distraction is limited by the tectorial and alar ligaments [13].

Therefore, according to the Anderson-Montesano classification, we believe that the mechanism of fracture in our patients classified in type I Anderson-Montesano classification was flexion. In contrast, in type III, it was a rotational mechanism with lateral flexion, as confirmed by Hanson [3]. Simultaneously on MRI in our OCF patients, we have not observed any type I Anderson-Montesano injury to the tectorial membrane and type III injury to the tectorial and alar ligaments. The authors agree with Tuli [22] that the C0–2 complex should be treated homogeneously in the assessment of OCF stability, as confirmed by our patient R.M., whose OCF was accompanied by a C1 fracture. This resulted (despite a type I OCF according to the Anderson-Montesano classification) in the patient’s qualification for treatment with the halo-vest according to Tuli’s type IIB fracture assessment. In contrast, evaluation of the C0–2 ligamentous apparatus in patients with OCF showed no traumatic damage except for avulsion fractures of the OCF.

While evaluating the outcomes of OCF in our patients based on the Anderson-Montesano and Tuli classifications, in our group of patients, the mean age was 15.8 years. We have tried to evaluate the use of these classifications in OCF in other authors’ research as well, especially in preadolescent patients. Although the youngest patient with OCF based on the bibliography was 7 months old [16], most of the descriptions of patients under 12 years of age were case reports [4, 10, 19, 23, 24], and they did not explicitly refer to the growth of the child during this period of life.

Nevertheless, the linear growth in childhood proceeds from birth to adolescence, with various intensities depending on the period of life. After the growth, spurt related to puberty, the height velocity decelerates and almost ceases due to epiphyseal fusion, typically at the skeletal age of 15 years in girls and 17 years in boys. However, the complete cessation of the growth, defined as four successive 6-monthly increments, each less than 0.5 cm, occurs at the age of 18.5 years in girls and 19.5 years in boys [25]. The length of the leg after the epiphyseal fusion is stable, but the small increment is still observed in sitting height, meaning that for the last couple centimeters of height increase, the increase in spinal cord length is responsible [26, 27]. Also the morphometric parameters of the cranio-cervical junction change during the whole childhood [28]. As Bapuraj et al. showed, the evolution of the cranio-cervical junction does not stop with the intense growth cessation, but proceeds to the age of 18 years, as the spinal cord is still growing. However, these morphological changes related to age do not lead to major changes in anatomical proportions of the junction and do not generate the need for the new classification formulation of occipital condyle fractures in children. It seems that the classification used for the adult can be suitable for adolescents’ occipital condyle fractures.

In summary, based on our series of pediatric patients with OCF, we did not observe differences and difficulties when evaluating OCF and qualifying for conservative or surgical treatment. And thus, we found no need to modify the Anderson-Montesano and Tuli classifications in pediatric patients treated for OCF.

### Limitation

Our pediatric patient’s group treated for OCF is not large (6 patients), and the patients are above 14 years of age. Different treatment modalities were also applied to those patients, based on the subjective assessment of OCF stability performed by the team of radiologists and orthopedists.

## Conclusion

1. The Anderson-Montesano and Tuli’s classifications of OCF can be used to assess the stability of OCFs in adolescents, but both classifications should be used simultaneously.
2. CT and MR imaging should be used in diagnosing OCFs, whereas CT allows assessing therapeutic outcomes in OCFs.

## Data Availability

Data and materials are available in Upper Silesian Child Centre in Katowice, Poland

## Abbreviation

OCF: Occipital condyle fracture
CCJ: Cranio-cervical junction
CT: Computed tomography
MRI: Magnetic resonance imaging
AOD: Atlanto-occipital dislocation
